# Characterization of Powder Bed Fusion–Laser Beam Ti6Al4V Samples in the As-Built and Stress-Relief States

**DOI:** 10.3390/ma19132888

**Published:** 2026-07-06

**Authors:** Paola Leo, Gilda Renna, Andrea Amleto De Luca, Chiara Scaramuzzi, Neetesh Soni, Francesco Willem Panella, Teresa Primo, Gabriele Papadia

**Affiliations:** Department of Innovation Engineering, University of Salento, 73100 Lecce, Italy; gilda.renna@unisalento.it (G.R.); andreaamleto.deluca@unisalento.it (A.A.D.L.); chiara.scaramuzzi@unisalento.it (C.S.); neetesh.soni@unisalento.it (N.S.); francesco.panella@unisalento.it (F.W.P.); teresa.primo@unisalento.it (T.P.); gabriele.papadia@unisalento.it (G.P.)

**Keywords:** Ti6Al4V alloy, microstructure of titanium alloy, stress-relief heat treatment, residual stress, Powder Bed Fusion–Laser Beam (PBF-LB), selective laser melting (SLM)

## Abstract

Despite the advantages of powder bed fusion–laser beam (PBF-LB), Ti6Al4V components often exhibit high yield strength but limited ductility, which restricts their use in critical structural applications. This study aims to identify the most effective heat treatment to optimize the strength–ductility balance in Ti6Al4V parts produced by PBF-LB and to establish direct correlations between microstructural states, mechanical properties and corrosion behavior. Two distinct post-processing heat treatments were applied, specifically, the first at 500 °C for 5 h and the second at 800 °C for 2 h, both followed by air cooling. The microstructure was characterized using optical microscopy (OM), scanning electron microscopy (SEM), and X-ray diffraction (XRD). Mechanical behavior was assessed through Vickers microhardness testing and tensile testing, while corrosion resistance was evaluated via electrochemical measurements. Residual stress profiles were determined using the hole-drilling strain gauge method, in both as-built and heat-treated conditions. The as-built samples displayed a fully martensitic α′ structure with columnar grains aligned parallel to the laser scanning direction, resulting from rapid solidification. Heat treatment at 500 °C caused only partial decomposition of acicular martensite into substructures without altering its acicular morphology, leading to a strengthening effect alongside a reduction in ductility. Conversely, heat treatment at 800 °C offered the most balanced combination of strength and ductility among the conditions studied, albeit with a moderate reduction in corrosion resistance.

## 1. Introduction

Titanium alloys can develop a variety of microstructural morphologies, including Widmanstätten, lamellar, basket-weave, equiaxed, martensitic and bimodal structures. The development of these microstructures is governed by the heat-treatment schedules as well as by the specific processing route adopted.

Recognized as the most widely used titanium alloy, Ti6Al4V currently accounts for nearly half of the global titanium market. Originally developed in the 1950s for aircraft structural applications [1], it remains predominantly employed in the aerospace sector, although its use has progressively expanded to the marine, automotive, energy, chemical, and biomedical industries over the past decades [2,3]. Its outstanding combination of high specific strength, excellent corrosion resistance, and good performance at moderately elevated temperatures makes it particularly suitable for heavily loaded aerospace components, where weight reduction and structural reliability are critical requirements [4]. For example, titanium can effectively replace aluminum when operating temperatures exceed approximately 130 °C, which is generally considered the maximum service temperature for conventional aluminum alloys [5].

**Figure 1:**
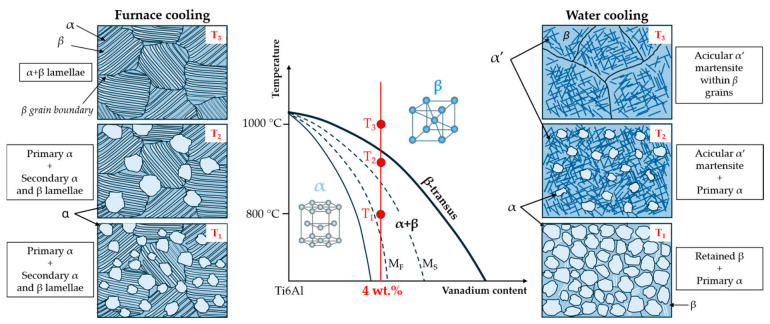
Schematic illustration of the different microstructures developed after heat treatments at temperatures below (T_1_ and T_2_) or above (T_3_) the β-transus, followed by furnace cooling and water quenching.

Ti6Al4V is an α+β titanium alloy characterized by a two-phase microstructure consisting of the hexagonal close-packed (hcp) α phase and the body-centered cubic (bcc) β phase, stable at low and high temperatures, respectively [6,7]. The transformation behavior of this alloy is governed by the β-transus temperature (≈985–1000 °C [8,9,10]), which marks the boundary between the α+β and single β phase fields (Figure 1).

A wide variety of heat treatments are used to control the phase fractions and morphology of the α and β phases, thereby tailoring the mechanical properties of Ti6Al4V to meet specific application demands [11,12].

The final microstructure is strongly influenced by the temperature range, holding time, and especially the cooling medium and cooling rate employed [13]. Figure 1 schematically illustrates the microstructural evolution of Ti6Al4V as a function of heat treatment temperature within the α+β or β phase fields and the applied cooling conditions. Above the β-transus temperature (T_3_), rapid quenching promotes martensitic transformation, whereas slower cooling rates allow diffusional β-to-α transformation, resulting in partial or complete lamellar α+β structures. Starting from the α+β phase field (at temperatures T_1_ and T_2_), rapid cooling leads to the retention of primary α grains and partial martensitic transformation of the remaining β phase. Conversely, furnace cooling favors equiaxed primary α grains and an intergranular β phase, along with a lamellar α+β morphology (bimodal microstructure) [14].

For conventionally processed Ti6Al4V alloys, such as wrought or forged products, common heat treatments include stress relieving, annealing, solution treatment and ageing, and β annealing, all of which are employed to achieve an optimal balance between strength and ductility [14]. Nevertheless, these manufacturing routes are often limited by high costs, significant material waste from machining, and restricted design freedom for complex geometries. These drawbacks have stimulated strong interest in additive manufacturing as a more sustainable and flexible alternative [2,3]. Among the various additive manufacturing processes, powder-bed fusion techniques such as PBF-LB are widely adopted to produce dense metallic components [4]. This process allows the layer-by-layer fabrication of parts directly from 3D CAD models by selectively melting metal powder with a laser beam, enabling the creation of complex geometries without the need for dedicated tooling or extensive post-processing [15,16]. Consequently, the combination of titanium alloys and PBF-LB technology has emerged as a highly promising solution, offering the possibility to produce lightweight components with high strength-to-weight ratios while minimizing material waste [17].

In the aerospace sector, this synergy enables significant weight reduction in heavily loaded structures, making titanium processed by PBF-LB particularly suitable in the production of jet engine components, gas turbines, and various airframe structures [18].

While PBF-LB-fabricated Ti6Al4V components typically exhibit higher strength than their conventionally manufactured counterparts, achieving an appropriate balance between ductility and fracture toughness remains a significant challenge for structural applications [4]. The PBF-LB process is governed by complex multi-physics and non-equilibrium (rapid solidification) phenomena, which are highly sensitive to a large number of parameters, including laser power, scan speed, scan strategy, layer thickness, hatch spacing, and powder characteristics [4,17]. Frequently, PBF-LB parts exhibit typical defects, high surface roughness, and substantial residual stresses, all of which can negatively impact mechanical performance [4,11,15]. Common process-induced defects include lack of fusion, gas entrapment, and delamination between adjacent layers or scan tracks [19]. Surface roughness represents another critical issue in as-built AM components [15]. For instance, Vayssette B. et al. [15] demonstrated a significant reduction in high-cycle fatigue strength in as-built Ti6Al4V specimens compared with machined ones. Furthermore, the highly localized heat input and short laser–material interaction time characteristic of PBF-LB generate steep thermal gradients and extremely high cooling rates (about 10^6^ K/s) [11,20]. These conditions induce significant residual stresses in Ti6Al4V components, which adversely affect fatigue resistance by facilitating crack initiation and propagation [4,21,22]. In particular, Parry L. et al. [23] reported that residual stresses are significantly higher along the scan direction than in the perpendicular direction, owing to the larger thermal gradients experienced during processing. This results in pronounced anisotropic stress distributions within the final part.

The rapid solidification associated with PBF-LB also has a major influence on microstructure formation. During the layer-by-layer melting and solidification process, steep thermal gradients promote epitaxial growth of prior-β columnar grains along the build direction, while the high cooling rate suppresses diffusional β → α+β transformation. As a result, as-built PBF-LB/Ti6Al4V generally exhibits a fine acicular α′ martensitic microstructure within prior-β columnar grains [3,4,19,20]. This metastable α′ martensite is characterized by solute supersaturation, high dislocation density, and fine lath morphology. These features are mainly responsible for the high strength and hardness commonly reported for as-built PBF-LB/Ti6Al4V, but they also contribute to limited ductility [3,9,19,20].

Therefore, post-processing heat treatments are essential to overcome these limitations. Extensive research has explored the effects of different post-processing strategies on additively manufactured Ti6Al4V [9,10]. Stress relief is commonly performed between 400 and 800 °C to reduce residual stresses and distortions [24,25]. Annealing in the range of 700–940 °C promotes martensite decomposition toward a bimodal microstructure, improving the strength–ductility balance [26]. Solution heat treatment above the β-transus, often followed by artificial aging at temperatures ≤ 700 °C, enables further microstructural optimization [27,28]. In addition, hot isostatic pressing (HIP), a thermo-mechanical treatment conducted at 900–1050 °C under isostatic pressures of ~100 MPa or higher, effectively eliminates process-induced porosity and lack-of-fusion, significantly enhancing ductility and fatigue resistance [29].

From a metallurgical point of view, the response of PBF-LB/Ti6Al4V to post-processing heat treatments is strongly related to the metastable nature of the α′ martensite formed during rapid solidification. It is well established that α′ martensite may progressively decompose or transform into more stable α+β phases, with the transformation behavior being governed mainly by the heat-treatment parameters, particularly the isothermal holding temperature, dwell time, and subsequent cooling condition [9,12]. Excessive temperatures or prolonged holding times may promote α-lath coarsening and microstructural over-aging, ultimately reducing the load-bearing capability and strength of the alloy [26,28].

Building upon these findings, several studies have investigated the effectiveness of specific heat treatment strategies. For example, Frkan M. et al. [30] investigated stress-relief treatments at 740 °C and 900 °C for 2 h, reporting slightly higher fatigue strength in samples treated at 900 °C, which was attributed to improved ductility. Similarly, Young-Kyun Kim et al. [31] investigated stress-relieved PBF-LB/Ti6Al4V heat-treated at 650 °C for 3 h followed by furnace cooling, demonstrating that the heat treatment eliminated hardness anisotropy and resulted in mechanical properties comparable to or exceeding those of wrought materials. Moreover, they showed that although the as-built samples exhibited higher yield strength, they displayed limited deformation and premature fracture, whereas the heat-treated specimens demonstrated improved ductility and greater deformation capability. Additionally, Eshawish N. et al. [32] observed that while the α′ phase remained dominant after treatment at 704 °C, significant microstructural transformations and improved fatigue performance occurred after higher-temperature treatments combined with controlled cooling.

Although post-processing heat treatments at high temperatures (>800 °C) and low temperatures (450–600 °C) are well documented as distinct metallurgical responses in AMed Ti6Al4V [7,9,33,34,35,36], their influence on the coupled evolution of mechanical and electrochemical properties has not been fully established. In particular, the interplay between the extent of α′ martensite decomposition, the resulting mechanical trade-off between strength and ductility, and the associated corrosion response remains only partially understood within a unified framework. As a result, most existing studies tend to evaluate mechanical performance and corrosion resistance separately, rather than as interdependent outcomes of the same thermal history.

To address this gap, the present study investigates two representative heat-treatment conditions, 500 °C and 800 °C, selected to represent distinctly different metallurgical states. The 500 °C treatment is employed as a stress-relief condition; in the 450–600 °C range, the thermal energy primarily promotes residual stress relaxation through recovery mechanisms and early-stage dislocation annihilation, while largely preserving the fine acicular α′ martensitic microstructure formed during rapid solidification [7,9,33]. Under these conditions, solute redistribution is limited and no significant phase transformation occurs, allowing the as-built high-strength state to be substantially retained. In contrast, the 800 °C treatment is intended to promote the progressive decomposition of α′ martensite into the equilibrium α+β microstructure [26,33,34,35,36]. As diffusion becomes increasingly active, vanadium partitioning from the supersaturated α′ lattice facilitates the nucleation and growth of β phase along α/α′ interfaces and plate boundaries [33]. Although this microstructural evolution is typically accompanied by a reduction in hardness and strength compared with the as-built condition, it generally results in a significant improvement in ductility [36].

Through a comparative analysis of the as-built condition and two post-processed states, combining microstructural characterization, residual stress assessment, mechanical testing, and electrochemical corrosion evaluation, this study aims to establish correlations between heat-treatment-induced microstructural states and their corresponding mechanical and corrosion performance in additively manufactured Ti6Al4V.

## 2. Materials and Methods

**Figure 2:**
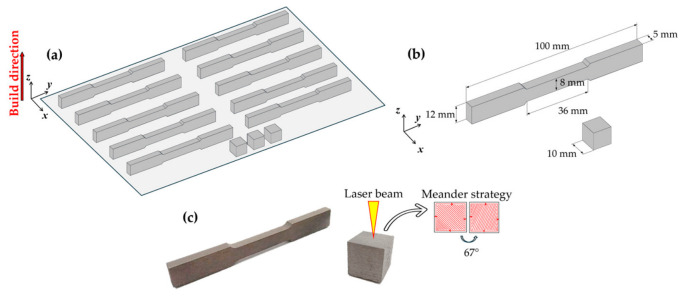
(**a**) Schematic representation of the cubic and dog-bone Ti6Al4V samples fabricated by PBF-LB on the build platform; (**b**) dimensions of the samples; and (**c**) images of the fabricated samples and a schematic illustration of the Meander laser scanning strategy, with a 67° rotation between successive layers. The building direction was parallel to the z-axis.

Gas-atomized Ti6Al4V powder was used to process cubes (10 mm edge) and dog-bone tensile test samples (Figure 2a,b) according to ASTM E8/E8M-22 [37]. The chemical composition of the powder is reported in Table 1. The samples were built by PBF-LB using a Renishaw AM 400 machine (Renishaw plc, Wotton-under-Edge, UK), equipped with a standard 250 × 250 × 40 mm build platform made of titanium alloy. During the build process, the build platform temperature was maintained at 170 °C. A Meander scan strategy with a 67° rotation between consecutive layers was applied (Figure 2c) to reduce thermal gradients and mitigate residual stress accumulation [38]. The processing conditions were defined according to a previously validated parameter set. Specifically, a volumetric energy density (VED) of 66 J mm^−3^ was employed, calculated according to Equation (1) as

(1) $$VED=\frac{P}{v\cdot t\cdot h}\;\left[J\;{mm}^{-3}\right]$$

where P, v, t, and h denote the laser power (400 W), scanning speed (1340 mm/s), layer thickness (0.060 mm), and hatch spacing (0.075 mm), respectively. Both the cubic and dog-bone samples were manufactured with the build direction parallel to the *z*-axis. After fabrication, the samples were removed from the build platform by wire electrical discharge machining (wire EDM). No subsequent machining or surface finishing operations were performed on the sections. This approach was deliberately adopted to preserve the as-built surface condition, allowing evaluation of the influence of surface roughness and residual stress on the mechanical properties and XRD results before and after heat treatments.

**Figure 3:**
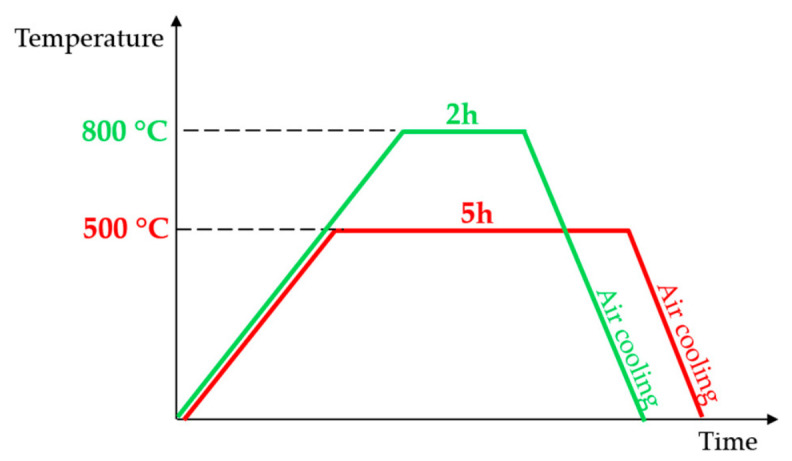
Schematic illustration of the thermal cycles used in the present work: holding at 500 °C for 5 h (HT1_500) and at 800 °C for 2 h (HT2_800).

The samples were subsequently subjected to two sub-transus heat treatments, which were specifically designed to produce different microstructural states and thereby investigate the influence of the treatments on the strength–ductility balance of the Ti6Al4V alloy. The thermal cycles applied are schematically illustrated in Figure 3. The first thermal cycle, hereinafter referred to as HT1_500, was designed to effectively promote the relaxation of residual stresses with minimal alteration of the fine acicular α′ martensitic microstructure typical of the as-built condition. Specifically, the samples were heated from room temperature to 500 °C at a controlled heating rate of 2.8 °C/min. The samples were subsequently held isothermally at 500 °C for 5 h.

The second thermal cycle, denoted HT2_800, was selected to promote a more pronounced microstructural transformation involving the decomposition of the metastable α′ martensite into a more stable α+β structure. Samples were heated to 800 °C at a rate of 4.5 °C/min and maintained at this temperature for 2 h. Both heat treatments were performed in a Lenton UAF 16/10 conventional electric muffle oven (Lenton Furnaces & Ovens, Parsons Lane, UK). The heating rates were selected to ensure thermal homogeneity throughout the sample volume and to minimize localized thermal expansion gradients that could interact with the high residual stresses inherited from the PBF-LB process. Nevertheless, the heating rate was not considered an independent experimental variable in the present study, and the observed microstructural evolution is therefore primarily attributed to the selected holding temperatures and soaking times.

At the end of the respective soaking periods, all samples were removed from the furnace and allowed to cool to room temperature under still-air conditions. Air cooling was selected to avoid the reintroduction of thermal macro-residual stresses typically associated with rapid quenching media.

The microstructures of the samples in the as-built (AB) and heat-treated states (HT1_500 and HT2_800) were characterized by a Nikon Ephipot 200 optical microscope (OM, Nikon, Tokyo, Japan) and a high-vacuum Zeiss EVO scanning electron microscope (SEM, Zeiss, Oberkochen, Germany) operated at an accelerating voltage of 20 kV. Metallographic samples for OM and SEM observations were first ground, using SiC grinding papers, up to #2000 grit size, then polished using a Al_2_O_3_ suspension, and finally etched by a modified Kroll’s reagent (95mL H_2_O, 4mL HNO_3_, 2mL HF). Porosity analysis was performed by optical microscope and utilizing ImageJ software (1.54G version). Defect morphology and size were quantified in terms of circularity and Feret diameter, respectively.

Surface roughness of the PBF-LB/Ti6Al4V samples was assessed, in accordance with ISO 4287:1997 [39], using a MarSurf PS 10 portable contact profilometer (Mahr GmbH, Göttingen, Germany). Measurements were performed with a cutoff length of 0.8 mm and an evaluation length of 4 mm. The roughness parameters Ra and Rz were determined, where Ra represents the arithmetic mean of the absolute profile deviations from the mean line, and Rz corresponds to the average height difference between the five highest peaks and the five deepest valleys within the sampling length.

X-ray diffractometry by a Rigaku D/MAX-Ultima X-ray system (Rigaku Holdings Corporation, Tokyo, Japan) with Cu (Kα) X-radiation (λ = 1.5406 Å) was used to further characterize the samples. The measurements were conducted on the *yz* section, applying a voltage and current of, respectively, 40 kV and 30 mA, in the 2θ range from 30–95°, with a scanning speed of 0.02° and an acquisition time of 1 s.

Vickers microhardness measurements were performed in the *xz* plane using an AFFRI Wiki 200 JS hardness tester (Affri, Varese, Italy), applying a load of 500 gf with a dwell time of 15 s. Each average microhardness value is obtained from 10 measurements. Specifically, two indentation lines were performed at a spacing of 2000 µm, with five measurements taken along each line at intervals of 1000 µm.

For each investigated condition, dog-bone tensile samples were tested using a Instron 3528 tensile test machine (Instron, Norwood, MA, USA) up to 250 kN, to determine the mechanical properties. Tensile tests were performed according to ASTM E8M [37], and at a crosshead displacement rate of 0.5 mm/min. Displacements were measured using an extensometer with a 25 mm gauge length. The ultimate tensile strength (UTS) and elongation at fracture were determined from the obtained stress–strain curves. A total of five specimens per condition were tested to validate the consistency of mechanical data. Table 1 summarizes the mechanical properties of wrought Ti6Al4V alloy, including ultimate tensile strength (UTS), yield strength (YS), Vickers hardness (HV), and elongation at fracture (ε).

Residual stress measurements were carried out on dog-bone specimens, using the hole-drilling technique, in accordance with ASTM E837-01 [40]. The MTS3000 RESTAN automated system (SINT Technology s.r.l., Calenzano, Italy) was employed, using CEA-062UM-120 rectangular strain gauge rosettes (Micro-Measurements, Wendell, NC, USA), which were configured in a half-bridge arrangement to minimize the influence of thermal strains. The hole was introduced incrementally, using a drilling rate of 0.08 mm/min, following a stepped parabolic distribution, to a depth of 1.5 mm.

Finally, the corrosion behaviors of the samples in the three different conditions were evaluated along the *xz*-plane. Prior to the electrochemical tests, all sample surfaces were carefully ground up to #2000 grit, using SiC paper, in order to ensure a uniform surface condition and minimize any surface-related influence in the comparisons among the different specimens. Before conducting polarization measurements, the open circuit potential (OCP) was recorded for 1 h to allow stabilization of the electrochemical system. Potentiodynamic tests were performed in a conventional three-electrode configuration, where the specimen acted as the working electrode, an Ag/AgCl electrode was used as the reference, and a graphite rod served as the counter electrode. All experiments were carried out in a 3.5 wt.% NaCl aqueous solution. Polarization scans were performed at a rate of 2 mV s^−1^ over a potential window ranging from −1.5 to 1.5 V (vs. Ag/AgCl), using a Gamry Interface 1010 potentiostat (Gamry Instruments, Warminster, PA, USA). The polarization data were subsequently processed by means of Tafel extrapolation according to ASTM G59-23 [41], enabling the determination of the corrosion potential (*E_corr_*) and corrosion current density (*i_corr_*). The latter parameter provides a quantitative assessment of the metal dissolution kinetics and represents an indicator of the corrosion susceptibility of the material under the investigated conditions. In addition, the corrosion rate was determined using Faraday’s law, in accordance with the ASTM G102 [42].

**Table 1:** Chemical composition of the Ti6Al4V powder used for the fabrication of the investigated specimens and mechanical properties of the conventionally processed alloy.

Chemical Composition of Ti6Al4V Powder [wt.%]								
Ti	Al	V	Fe	O	C		N	H
Bal.	6.19	4.3	0.9	0.07	0.03		0.007	<0.001
**Mechanical properties of conventionally manufactured-Ti6Al4V**								
YS [GPa]		UTS [GPa]		ε [%]		HV [GPa]		Ref.
1.17–1.23 *		1.23–1.29 *		12–14 *		3.8–4.3 *		[43]

* The ranges of the mechanical properties are related to the different microstructures resulting from the various wrought processing conditions.

## 3. Results and Discussion

### 3.1. Characterization of As-Built Samples

In the PBF-LB process, the feedstock metal powder undergoes repeated melting–solidification cycles as a result of the layer-by-layer build-up and the track-by-track scanning sequence within each layer. These repeated thermal cyclIes lead to the development of anisotropic mechanical properties, the formation of metastable microstructures, and the accumulation of residual stress. In addition, the laser processing parameters play a critical role in determining the morphology and stability of the melt pool, which directly affect the surface finishing (in terms of roughness) and the density of the fabricated parts, particularly through the formation of defects. These features are of primary importance, as defects and surface irregularities may act as stress concentrators, thereby influencing the mechanical performance of the components. For this reason, the as-built samples were first characterized in terms of defect fraction and surface roughness to assess the overall quality of the printed parts. The defect fraction was determined as the ratio of the total void area to the overall area of the examined section. Contrastingly, surface roughness was quantified using the parameters Ra and Rz. Both analyses were performed on the *xy*-plane and *xz*-plane, oriented, respectively, perpendicular and parallel to the laser build direction.

**Figure 4:**
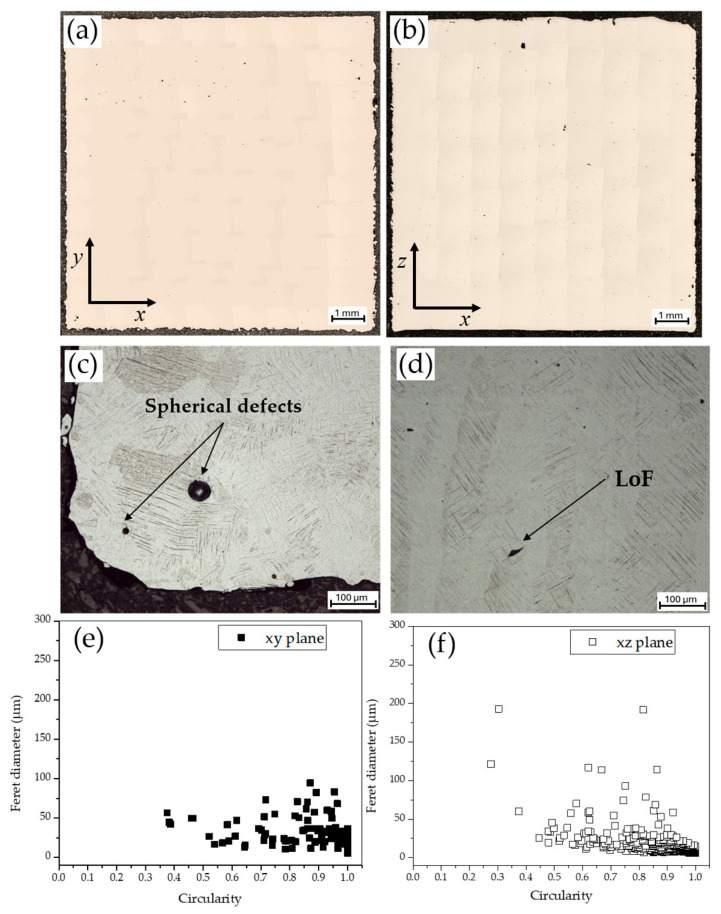
Porosity characterization in the as-built condition: optical macrographs of (**a**) *xy*-plane and (**b**) *xz*-plane before chemical etching; detailed high-magnification micrographs showing representative defects in (**c**) *xy*-plane and (**d**) *xz*-plane; circularity–Feret diameter graphs showing the distributions of defects for both the (**e**) *xy* and the (**f**) *xz* sections.

The quantitative defect analysis (Table 2) revealed high densification levels, along with a negligible porosity fraction. Specifically, the defect fraction was about 0.07% in the transverse (*xy*) section and 0.15% in the longitudinal (*xz*) section, indicating a nearly full-density consolidation. The corresponding optical macrographs of the entirety of the cross-sections (Figure 4a,b) confirm this low defect density in both planes, which is consistent with optimized PBF-LB process parameters. At higher magnification, the microstructure exhibits the typical defects commonly found in PBF-LB processed materials, namely, gas-induced spherical porosity (Figure 4c) and lack-of-fusion (LoF) defects (Figure 4d). These defect types are widely reported in the literature and are generally attributed to different physical mechanisms [44,45]. Gas-entrapped pores are generally small and spherical, resulting from gas entrapment (argon or evaporated elements) within the melt pool. Lack-of-fusion defects, on the other hand, appear more irregular and elongated, arising from insufficient energy input and incomplete melting between adjacent tracks or layers. Additional insight into the defect characteristics is provided by the scatter plots of circularity (shape factor) versus Feret’s diameter (Figure 4e,f), which allow differentiation between nearly spherical pores (circularity 0.8–1.0) and more irregular lack-of-fusion defects (circularity 0.3–0.8), based on their combined size and shape distributions. Specifically, in the *xy*-plane (perpendicular to the build direction), the defects are mostly small, with Feret diameters not exceeding 100 µm, and the majority exhibit high circularity values close to 1, indicating a predominantly spherical morphology. In contrast, the *xz*-plane (parallel to the build direction) shows a broader defect-size distribution, including some larger defects with Feret diameters up to approximately 200 µm. These larger defects are associated with significantly lower circularity values, consistent with their irregular and elongated shape, which is characteristic of lack-of-fusion defects.

**Figure 5:**
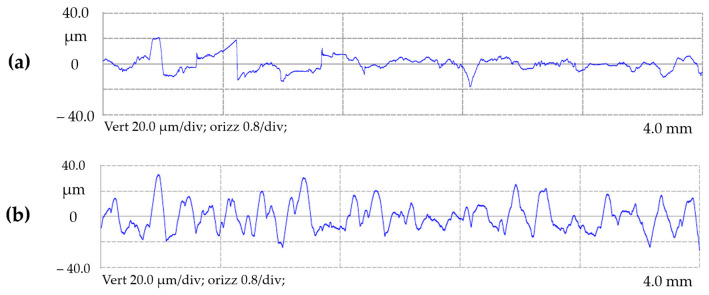
Surface roughness profiles of the PBF-LB-processed cubic specimen: (**a**) xy plane and (**b**) xz plane.

The slightly higher defect fraction observed in the *xz*-plane is consistent with the surface roughness measurements, which revealed a higher surface roughness in the *xz*-section compared to the *xy*-section (Table 2). The corresponding roughness profiles reported in Figure 5a and Figure 5b for the *xy*- and *xz*-sections, respectively, further highlight this difference, with the *xz*-plane exhibiting more pronounced surface texture (height fluctuations) and a less uniform surface morphology. This topographic anisotropy is a characteristic byproduct of the layer-stacking effect inherent to the PBF-LB process, where the interfaces between successive layers create a more pronounced surface profile along the build axis. Despite the presence of slightly higher surface roughness in the *xz*-section, the overall defect fraction remains extremely low, indicating that the selected processing parameters ensured stable melt pool formation and effective metallurgical bonding between adjacent layers.

**Figure 6:**
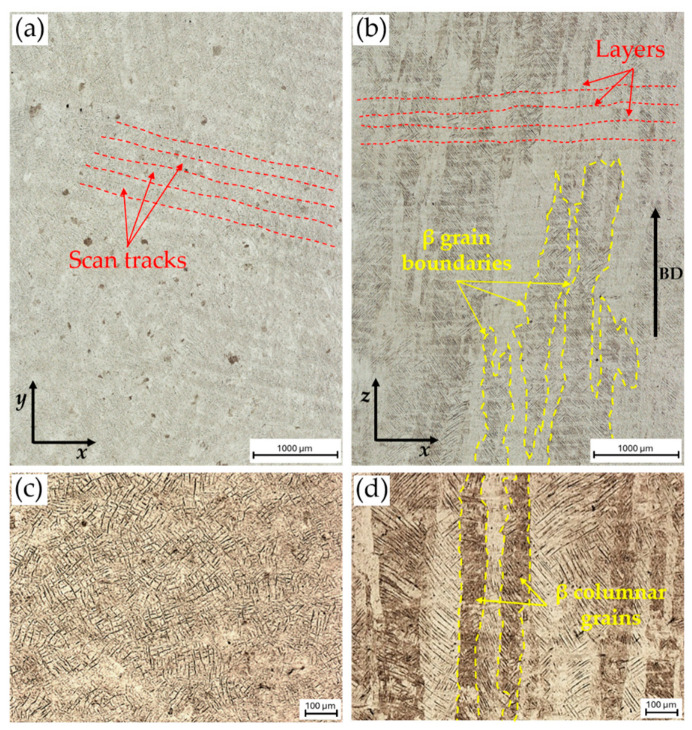
(**a**,**b**) OM macrographs of the as-built PBF-LB/Ti6Al4V sample and (**c**,**d**) micrographs acquired on the (**c**) *xy* plane and the (**d**) *xz* plane.

The resulting microstructures of the as-built samples, as observed in the two different sections, are reported in Figure 6. Specifically, Figure 6a and Figure 6b show optical macrographs of the *xy*- and *xz*-sections, corresponding to the perpendicular plane and parallel to the laser beam (and build direction), respectively. The red dashed lines highlight the laser scanning pattern in the *xy*-plane and the layer-by-layer microstructural arrangement in the *xz*-plane, illustrating the characteristic build strategy adopted in the PBF-LB process. In the section perpendicular to the laser beam (*xy*-plane), the microstructure is characterized by a predominantly acicular α′ martensitic phase (Figure 6a,c). This metastable phase forms as a result of the steep thermal gradients and high cooling rates (10^3^–10^6^ K/s) inherent to the laser melting process, which inhibit the diffusion-controlled transformation toward the equilibrium α+β phases. The resulting α′ martensite is characterized by a high concentration of vanadium in solid solution, elevated dislocation density [2,20], and occasional twinning. Such microstructural features act as strong barriers to dislocation motion, thereby providing high yield strength at the expense of overall ductility, which is typically below 10% in as-built Ti6Al4V components [9,46].

Conversely, the optical micrographs of the *xz*-plane (Figure 6b,d) reveal the presence of primary columnar β grains, highlighted in yellow, oriented parallel to the build direction. These grains exhibit epitaxial growth across successive deposited layers, often reaching lengths of several millimeters. The persistence of this columnar morphology is a distinctive microstructural feature of the PBF-LB process, arising from the maximum thermal gradient that guides the preferential solidification along the crystal growth direction. Within these large columnar β grains, a fine, needle-like martensitic α′ phase was observed, in agreement with previous reports in the literature [47,48,49].

A quantitative assessment of the prior-β grain morphology yielded an average columnar grain width of 168.3 ± 16 μm, closely corresponding to the laser scan track width (166 ± 1 µm). This close correspondence indicates that the selected scan strategy and volumetric energy density (VED = 66 J mm^−3^) directly governed the development of the resulting macrostructural grain architecture. Within this framework, the 67° scan rotation strategy employed between successive layers plays a relevant role in shaping the solidification structure by continuously altering the spatial orientation of adjacent melt pools and mitigating the directional accumulation of process-induced heterogeneities and defects at track overlaps. Nevertheless, while this interlayer rotation effectively limits lateral grain coarsening within the build plane, the dominant directional heat extraction toward the build platform remains the primary driving force governing the competitive epitaxial growth of prior-β grains along the build direction. Comparable prior-β grain sizes have been reported in the literature, although considerable variations have been observed as a function of processing parameters, thermal gradients, and solidification conditions. For instance, Vrancken B. et al. [50] and Simonelli M. et al. [51] reported average prior-β grain widths of approximately 55 ± 5 μm and 210 ± 50 μm, respectively, reflecting the sensitivity of grain growth to laser energy input and scan track overlap.

### 3.2. Heat Treatment-Induced Microstructural Changes

**Figure 7:**
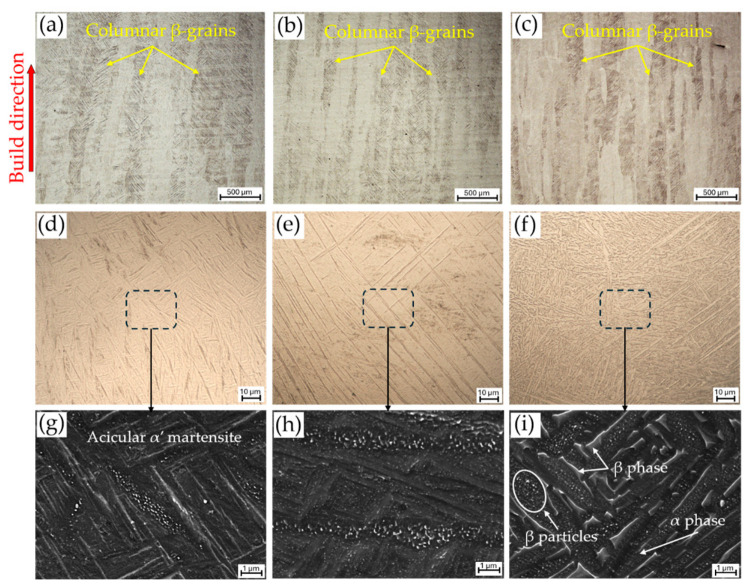
OM and SEM micrographs acquired on the *xz* plane of PBF-LB/Ti6Al4V samples in the as-built condition (**a**,**d**,**g**), after heat treatment at 500 °C (**b**,**e**,**h**), and after heat treatment at 800 °C (**c**,**f**,**i**).

In the present study, the different responses of the PBF-LB/Ti6Al4V microstructure to the two selected heat treatments are clearly reflected in the microstructural observations and XRD results. To assess the influence of post-processing heat treatments on PBF-LB-fabricated parts, detailed microstructural characterization combined with X-ray diffraction (XRD) analysis was performed on the *xz*-plane for both the as-built samples and the heat-treated ones. The resulting microstructural evolution is presented in Figure 7. Consistent with previous studies [50,52], both HT1 (500 °C) and HT2 (800 °C) were performed below the β-transus temperature. As a result, no substantial modification of the prior-β grain architecture was observed, and the characteristic columnar morphology together with the prior-β grain boundaries remained largely preserved after heat treatment. In addition, high-magnification SEM observations of the as-built condition reveal the presence of fine β-phase particles dispersed within the martensitic matrix (Figure 7a). These features are likely associated with the partial decomposition of α′ through diffusion-driven processes induced by the repeated thermal cycling occurring during the PBF-LB process [3]. Such microstructural characteristics contribute to enhanced hardness while simultaneously promoting a decrease in ductility, leading to a more brittle mechanical behavior.

Following heat treatment at 500 °C (Figure 7b), the microstructure remains predominantly martensitic, and no significant morphological changes can be clearly detected by optical microscopy (OM) or scanning electron microscopy (SEM), suggesting that the thermal energy at this temperature is insufficient for a complete phase transformation. Nevertheless, advanced transmission electron microscopy investigations reported in the literature [53] indicate that the martensitic plates undergo a process of elemental partitioning and in situ decomposition without losing their acicular morphology. Specifically, high-angle annular dark-field (HAADF) STEM imaging and selected area electron diffraction (SAED) patterns of samples treated at 500 °C reveal the emergence of parallel bright contrast traces and twin-like plates within the original α′ laths. These features, characterized by high Z-contrast, indicate a local enrichment of heavy elements (such as vanadium), confirming that solute redistribution is already active at this temperature. Although some works in the literature suggest the possible precipitation of intermetallic Ti_3_Al (α_2_ phase), SAED analysis in these studies often identifies these plates as still being the α phase, due to the absence of superlattice spots at the 1/2{010}α′ positions [53]. Previous studies on Ti6Al4V have shown that the driving force responsible for the formation of the α_2_ phase within the α matrix is controlled by the combined effects of aluminum and oxygen levels. In particular, oxygen atoms reduce the maximum solubility of aluminum within the α phase, thereby promoting the formation of Ti_3_Al precipitates [54].

In contrast, after heat treatment at 800 °C (Figure 7c), although this is still below the β transus temperature, a significant microstructural transformation takes place. The α′ martensite fully decomposes into a lamellar microstructure composed of α laths surrounded by a retained or reprecipitated β phase. So, this transformation leads to a more stable α+β microstructure, which is generally associated with improved ductility and a more balanced mechanical behavior compared to the as-built martensitic condition. In addition, a small number of nanoscale particles of β phase [55] can be observed dispersed on the α laths. As demonstrated by Zhang M. [56], in a comparative study of furnace and air cooling after heat treatments in the 800–900 °C range, the formation of these β nanosized particles is promoted by higher cooling rates. Under slower cooling conditions, diffusion-controlled processes enable their progressive dissolution.

**Figure 8:**
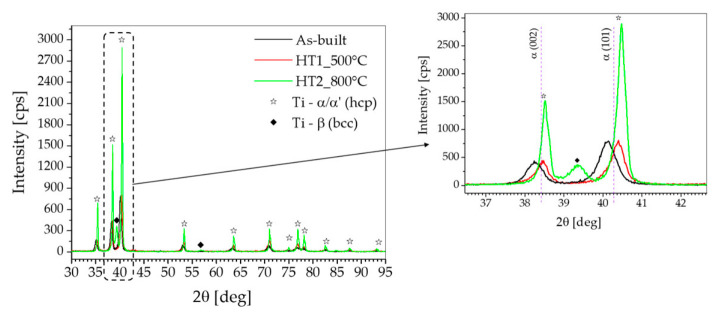
X-ray diffraction (XRD) patterns of the *yz* plane for the samples in the as-built condition and after heat treatments at 500 °C and 800 °C.

X-ray diffraction (XRD) analysis (Figure 8), corroborated by microstructural observations, provides additional insights into phase evolution and the internal stress state. The diffraction pattern of the as-built sample is characterized predominantly by α′ peaks. The sample heat-treated at 500 °C exhibits an almost identical diffraction pattern (α′ and/or α phase peaks). This observation is in agreement with the microstructural analysis (Figure 6), which showed that the martensitic structure remains largely preserved at this temperature, with only limited decomposition occurring at the nanoscale level, as reported in the literature [53]. The α and α′ phases exhibit the same hexagonal close-packed (HCP) crystal structure and very similar lattice parameters [57,58,59,60]. Their main difference arises from the diffusion-less β → α′ martensitic transformation, which retains vanadium in supersaturated solid solution and induces a slight lattice distortion. As a result, the diffraction peaks of the two phases largely overlap, making their reliable distinction by XRD analysis particularly challenging. Nevertheless, XRD analysis of the as-built and HT1_500 sample has not shown any phase different from the HCP crystal structure (Figure 8), which may be due to the low number of precipitated particles or to the detection limit of the instrument.

In contrast, the heat treatment at 800 °C leads to an increased β-phase content, in agreement with the findings reported by [56]. This is corroborated by the presence of the β-Ti (110) peak near 2θ = 39.5° in the XRD pattern, indicating that a measurable amount of β-Ti phase is retained in the microstructure at room temperature following the thermal cycle.

Furthermore, a general increase in the diffraction peaks’ intensities after heat treatment is observed, which can be attributed to the coarsening of the microstructure and the reduction of lattice defects and residual stresses induced by thermal exposure. The same phenomenon has been previously reported in the literature by [49,56]. To provide a deeper insight into lattice strain and phase evolution, the full width at half maximum (FWHM) of the primary HCP α/α′ diffraction peak, corresponding to the (101) plane, was quantitatively evaluated. The as-built sample showed a maximum FWHM value of 0.49°. According to Jovanović M.T. et al. [59] and Baghi A.D. et al. [61], FWHM values exceeding 0.20° are typically associated with a highly distorted martensitic α′ structure resulting from the rapid solidification conditions and high microstrain inherent to the L-PBF process. Following thermal treatment, a progressive reduction in FWHM was observed, decreasing to 0.36° at 500 °C and 0.23° at 800 °C. The intermediate value measured for the 500 °C condition suggests partial recovery of the martensitic microstructure, showing a reduction in defect density while retaining the acicular α′ morphology. The further decrease observed at 800 °C indicates a more advanced microstructural evolution toward a stable α+β configuration, accompanied by a reduction in lattice strain and lath coarsening (see Figure 7c).

One critical observation concerns the peak positions relative to the theoretical 2θ angles (see magnification of Figure 8). In the as-built state, the peaks are noticeably shifted toward lower 2θ angles, a phenomenon characteristic of lattice expansion induced by residual tensile stresses and the supersaturation of the hexagonal lattice with vanadium atoms. Following both heat treatments, a progressive shift toward higher 2θ values was detected. This shift indicates a reduction in the lattice d-spacing, which corresponds to the relaxation of internal stresses and the progressive restoration of the crystalline lattice toward its equilibrium configuration.

### 3.3. Residual Stresses Measurement

**Figure 9:**
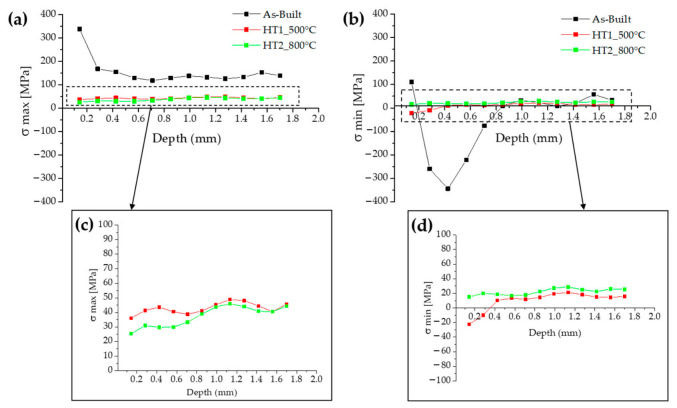
Residual stress distributions as a function of depth: (**a**) σ_max_ and (**b**) σ_min_ profiles for samples in the as-built condition and after HT1_500 °C and HT2_800 °C; magnification of (**c**) σ_max_ and (**d**) σ_min_ profiles for HT1_500 °C and HT2_800 °C samples.

Residual stress profiles, determined through the hole-drilling strain gauge method, are shown in Figure 9a–d, depicting the variations of the maximum (σ_max_) and minimum (σ_min_) principal stresses with depth. Holes were made along the symmetrical axis of the dog-bone samples, on the *yz*-plane, in the immediate vicinity of the fillet region. It can be observed that the as-built condition exhibits high tensile residual stresses at the surface. In particular, the σ_max_ surface stress reaches values of approximately 350 MPa and decreases rapidly within the first 0.4 mm below the surface (Figure 9a). At greater depths, the stress stabilizes in the range of about 120–150 MPa, even though there is some doubt about the reliability of deep data. Regarding the minimum principal stress (σ_min_), the stresses are initially tensile at the surface, with values close to 100 MPa (Figure 9b). However, a compressive peak of about −350 MPa is observed at a depth of around 0.4 mm, followed by a gradual increase toward lower absolute values. This behavior reflects the typical internal stress equilibrium characterized by the coexistence of tensile and compressive zones generated during the rapid thermal cycles of the additive manufacturing process. It is interesting to note that both heat treatments are effective in relieving the residual stresses, reducing them to negligible values in the investigated depth range. No substantial differences are observed in treated samples processed at 500 °C and 800 °C. Nevertheless, a more detailed examination of the heat-treated sample profiles (Figure 9c,d) suggests that the 800 °C treatment provides slightly more effective stress relaxation, which is likely linked to the microstructural transition from α′ martensite to the equilibrium α+β phases and the corresponding dislocation recovery observed in the XRD analysis.

### 3.4. Mechanical Behavior: Vickers Microhardness Measurement and Tensile Test

**Figure 10:**
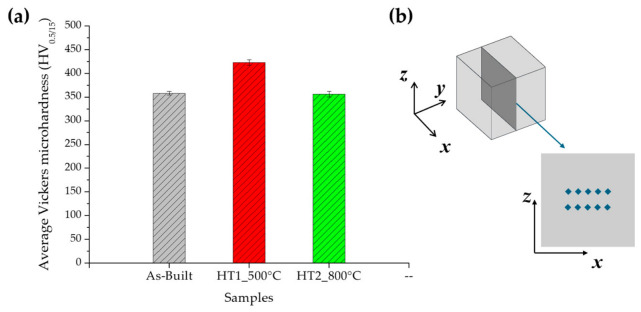
(**a**) Average Vickers microhardness values (HV_0.5/15_) measured on the *xz* plane for samples in as-built condition and after heat treatments at 500 °C and 800 °C; (**b**) schematic representation of the indentation pattern on the cubic sample.

**Figure 11:**
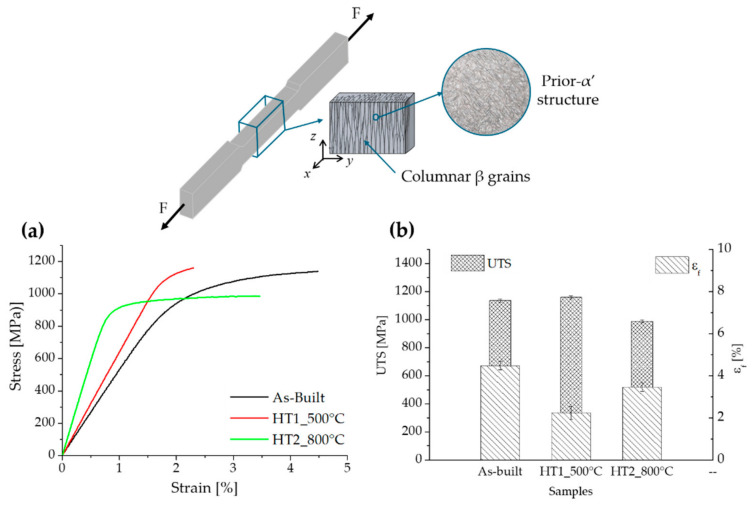
(**a**) Representative engineering stress–strain curves and (**b**) corresponding ultimate tensile stress (UTS) and elongation at fracture (ε_f_) values for the PBF-LB/Ti6Al4V samples evaluated in the as-built condition and after heat treatments at 500 °C (HT1_500 °C) and 800 °C (HT2_800 °C).

The average Vickers microhardness values (HV_0.5/15_) measured on the *xz* plane as a function of post-processing heat treatment are reported in Figure 10, whereas the corresponding tensile properties are presented in Figure 11. Specifically, Figure 11a displays representative engineering stress–strain curves of the PBF-LB fabricated Ti6Al4V samples in the as-built condition and after the different heat cycles. Complementing these curves, Figure 11b summarizes the corresponding ultimate tensile strength (UTS) and elongation at fracture (ε_f_), which offer critical insights into the material’s mechanical behavior and plastic deformation capacity. The observed trends are in close agreement with the phase transformations and stress-relief phenomena previously identified via XRD and residual stress analysis.

The comparative analysis reveals that the as-built condition achieves high hardness (358 ± 4 HV) and an elevated UTS (≈1134 MPa), which is associated with a ductility of ≈4.5%. This high strength is strictly linked to the fine acicular α′ martensitic microstructure and the high dislocation density characteristic of the rapid cooling rates inherent to the PBF-LB process. However, despite this high hardness, the as-built state remains susceptible to significant residual tensile stresses (as shown in Figure 9) that could compromise its fatigue resistance and structural stability.

Following the post-processing heat treatment at 500 °C for 5 h (HT1_500), a moderate hardness increase of approximately 50 HV was recorded. This hardening behavior aligns with the findings of Wu et al. [53], who reported a similar hardness rise around 500 °C; using TEM investigations, they attributed this phenomenon to the localized, in situ decomposition of the highly metastable α′ martensite into extremely fine, transitional substructures that remain unresolved under conventional OM and SEM examinations. Similarly, Zhang X.Y. et al. [48] observed an analogous strengthening effect at 600 °C, ascribing it to the partial decomposition of the martensitic microstructure and the resulting microstructural refinement.

As detailed in Section 3.2, thermal exposure at 500 °C may also promote the formation of the Ti_3_Al (α_2_) precipitates. Although direct evidence of α_2_ precipitation was not obtained in the present study, the formation of coherent α_2_ particles within the α matrix has been reported to contribute to increased yield strength, while simultaneously reducing tensile ductility. This detrimental effect on ductility arises from two main mechanisms: the generation of planar slip bands, which facilitate crack nucleation and propagation, thereby lowering fatigue resistance [62,63], and the pinning effect of the precipitates, which restricts dislocation glide, decreases the number of active slip systems, and ultimately limits the material’s capacity for plastic deformation and energy absorption.

Overall, although the heat treatment at 500 °C promotes residual stress relaxation, as evidenced by the XRD results and residual stress profiles (Figure 8 and Figure 9), the concurrent microstructural refinement and possible precipitation-hardening effects (coherent Ti_3_Al (α_2_) intermetallic) introduce additional barriers to dislocation motion [54]. Consequently, a slight increase in UTS is accompanied by a significant reduction in elongation to failure (ε_f_), resulting in a less ductile mechanical response compared with the as-built condition (Figure 11b).

Conversely, when the heat treatment temperature is raised to 800 °C (HT2_800), the hardness decreases markedly, returning to values similar to those of the as-built condition. This softening can be attributed to several concurrent mechanisms, including the progressive decomposition of the fine acicular α′ martensite into an equilibrium α+β microstructure, as well as the increase in α lath width and grain size, as reported by Zhang M. et al. [56]. The formation of a higher fraction of the softer β phase (relative to α phase) further contributes to the observed hardness reduction [54].

The tensile results in Figure 11 clearly demonstrate that the 800 °C treatment (HT2_800) is more effective than the 500 °C condition. By bypassing the embrittlement range associated with the α_2_ phase [49] and promoting the α′ → α+β transition, HT2_800 treatment replaces the high-dislocation martensitic matrix with a balanced dual-phase microstructure, thereby improving the material’s ductility (Figure 11b). However, as reported by Cao et al. [36], the 2 h holding time employed in the present study is insufficient to achieve complete martensite decomposition. This partial transformation enables the material to retain a considerable fraction of its strength. This optimizes the strength-to-ductility ratio, ensuring that the PBF-LB-produced Ti6Al4V can withstand high service-life loads without premature brittle failure.

Additionally, while interstitial oxygen absorption must be considered as a potential embrittling [46] factor in both treatments, the structural benefits of the 800 °C treatment successfully counteract these effects, yielding a material with enhanced overall toughness.

Beyond microstructural evolution induced by heat treatment, the mechanical response reported in Figure 11 is also significantly influenced by the inherent anisotropic architecture of the PBF-LB process. To properly interpret the trends observed in the engineering stress–strain curves (Figure 11a) and in the corresponding UTS and elongation values (Figure 11b), the influence of the anisotropic prior-β columnar grain architecture must be considered. As discussed in Section 3.1, the rapid solidification conditions and steep thermal gradients characteristic of the PBF-LB process promote epitaxial growth of prior-β grains along the build direction (*z*-axis). Since the tensile specimens were manufactured horizontally on the build platform (Figure 2), the applied tensile load was oriented perpendicular to the major growth direction of the prior-β columnar grains.

When loading acts perpendicular to the elongated prior-β grain boundaries, these macroscopic interfaces are subjected to severe normal separation stresses. Under these conditions, the continuous boundaries behave as structural weak links, where local manufacturing defects, un-melted powder particles, or residual pore networks, often located at laser scan track overlaps, can rapidly evolve into critical stress concentrators [64,65]. Upon plastic yielding, these transverse interfaces promote early micro-void nucleation, rapid coalescence, and localized inter-lath decohesion [66]. As a result, plastic deformation is reduced compared with vertically built and longitudinally loaded counterparts, as widely reported in the literature [61,64,65,66].

Importantly, this underlying macrostructural anisotropy is fully retained across all investigated post-processing conditions. Because both the HT1 heat treatment at 500 °C and the HT2 heat treatment at 800 °C are sub-transus thermal treatments, they lack the thermodynamic driving force required to trigger recrystallization or significant modification of the prior-β grain morphology [50,56]. Consequently, while thermal exposure effectively drives strains and partial α′ decomposition at 500 °C (0.36° FWHM) and promotes diffusion-controlled decomposition into a stable α+β mixture at 800 °C (0.23° FWHM), the primary columnar β architecture remains structurally unaltered (Figure 7). The mechanical performance of the heat-treated alloy is therefore governed by a competitive interplay between intra-granular microstructural optimization and the persistent, defect-sensitive anisotropic skeleton inherited from the PBF-LB solidification process.

### 3.5. Corrosion Behavior

**Figure 12:**
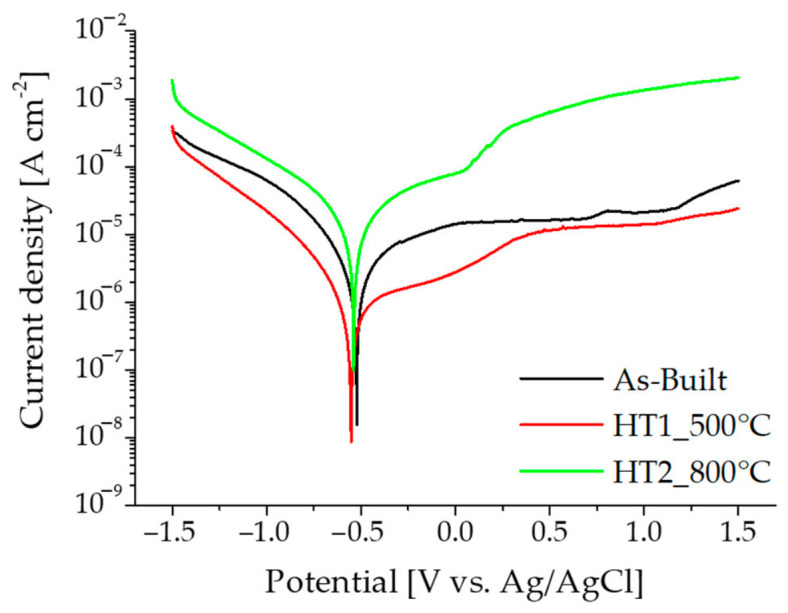
Potentiodynamic polarization curves of the as-built samples and the samples heat-treated at 500 °C and 800 °C, measured on the *xz* plane.

Finally, the corrosion response of the Ti6Al4V samples in the as-built and heat-treated conditions was characterized by potentiodynamic polarization measurements, with the corresponding curves as presented in Figure 12. The electrochemical parameters (*E_corr_* and *i_corr_*) extracted from the linear portions of the anodic and cathodic branches of the potentiodynamic polarization curves are summarized in Table 3. The corrosion potential (*E_corr_*) is defined as the potential at which no external current is applied, and the anodic and cathodic reaction rates are equal. At this point, the metal corrodes at a rate defined by the corrosion current density, *i_corr_* [67]. In accord with ASTM G102 [42], *i_corr_* is commonly used as an indicator of corrosion kinetics and can therefore be employed to estimate the corrosion rate of metallic materials. Generally, lower values of corrosion current density correspond to slower corrosion processes and, consequently, to improved corrosion resistance. When evaluating the corrosion behavior on the section parallel to the laser beam scanning (*xz*-plane), it can be observed that *E_corr_* values exhibited minimal variation among the three conditions, ranging between −0.522 V and −0.553 V vs. Ag/AgCl, suggesting that the thermodynamic susceptibility necessary to initiate corrosion remains largely unaffected by the thermal treatments. However, a significant difference was observed in the corrosion current density. The as-built sample displayed an *i_corr_* of 4.46 × 10^−6^ A/cm^2^, representing the baseline corrosion behavior. This observed value is attributed to the combined presence of high tensile residual stresses on the surface sample (Figure 9a) and the metastable α′ martensitic microstructure (Figure 7a). The role of martensitic microstructures in corrosion resistance is still a subject of debate in the literature.

Some authors report that the martensitic microstructure adversely affects corrosion resistance [68,69], whereas others suggest a beneficial effect [70].

In the last case, the improved corrosion resistance is attributed to the enhanced stability of the naturally formed TiO_2_ passive film on the surface, which is promoted by the homogeneous nature of the martensitic phase and the retention of alloying elements in solid solution [71].

Notably, in the present study, the authors agree that the presence of a martensitic microstructure leads to a lower degradation rate of Ti6Al4V components. However, the presence of tensile residual stresses at the sample’s surface may locally weaken this protective TiO_2_ film.

The sample heat-treated at 500 °C for 5 h exhibits the highest corrosion resistance, with *i_corr_* decreasing significantly to 8.08 × 10^−7^ A/cm^2^, approximately one order of magnitude lower than the values measured for the other conditions. Since the microstructure remains essentially unchanged (Figure 7b), this improvement can mainly be attributed to the relief of residual stresses induced by the heat treatment, which may reduce the driving force for localized corrosion phenomena. Consistently, this condition also exhibits the lowest corrosion rate (0.007 mm/year), confirming its superior electrochemical stability.

In contrast, the sample treated at 800 °C for 2 h shows a comparatively higher corrosion current density (*i_corr_* equal to 1.21 × 10^−5^ A/cm^2^). Similarly, Nianwei Dai [49] found that heat treatment at 850 °C leads to an increase in corrosion current density, thereby deteriorating the corrosion resistance. Although this higher temperature also promotes stress relaxation, microstructural coarsening and the increased volume fraction of the β phase have a detrimental effect on corrosion performance. In Ti6Al4V, the β phase is often more susceptible to localized attack, due to its higher vanadium content [47], which can create micro-galvanic couples with the surrounding α-matrix, thereby accelerating the dissolution kinetics [72]. As a result, the 800 °C condition exhibits the highest corrosion rate among the investigated states (0.104 mm/year), resulting in corrosion performance even worse than that of the as-built condition.

## 4. Conclusions

The present work provides a thorough evaluation of how PBF-LB processing, in combination with tailored post-heat treatments, influences the microstructure, residual stresses, mechanical performance, and corrosion behavior of Ti6Al4V components. The correlations established between microstructural changes and mechanical properties offer key guidelines for optimizing the performance of additively manufactured titanium alloys. Based on the experimental evidence, the following conclusions can be drawn:

- The as-built components exhibit a characteristic, metastable, fully acicular α′ martensitic microstructure and high densification (porosity < 0.15%). Heat treatment at 500 °C maintains this martensitic morphology while inducing only partial decomposition of the martensite and may additionally promote secondary hardening effects due to the formation of Ti_3_Al. In contrast, the 800 °C treatment promotes the equilibrium α′ → α+β phase transformation and microstructural coarsening.
- A direct correspondence was established between the laser scan track width and the prior-β columnar grain width (168.3 ± 16 μm), highlighting the dominant role of the processing parameters in governing the resulting macrostructural features. This columnar architecture remains essentially preserved by sub-transus heat treatments at 500 °C and 800 °C
- Quantitative XRD analysis revealed a progressive reduction in the full width at half maximum (FWHM) of the primary HCP peak, decreasing from 0.49° (as-built) to 0.36° (500 °C) and 0.23° (800 °C), indicating a systematic reduction in lattice strain with increasing thermal exposure. At 500 °C, the intermediate FWHM value suggests significant stress relaxation through dislocation recovery while largely preserving the acicular α′ martensitic morphology. In contrast, the 800 °C condition reflects a more advanced microstructural evolution toward a stable α+β configuration.
- The PBF-LB process induces significant surface tensile residual stresses (up to 350 MPa). Both the 500 °C and the 800 °C treatments are highly effective in achieving near-complete stress relief.
- The 500 °C treatment leads to a secondary hardening effect (+50 HV) but causes a drastic reduction in ductility (50.2%), likely due to the formation of substructure and precipitation of Ti_3_Al intermetallic. In contrast, the 800 °C treatment leads to limited reductions in ductility (22.6%) and energy absorption capacity (13%), resulting in the most favorable balance between strength and ductility among the investigated conditions.
- While the 500 °C treatment yields the lowest corrosion rate (0.007 mm/year), the 800 °C treatment exhibits a slight increase in corrosion kinetics, which was attributed to the enhanced formation of the β phase and coarsening of the microstructures.

In sum, although a slight decrease in corrosion resistance is observed, heat treatment at 800 °C can be considered the optimal post-treatment condition for Ti6Al4V components manufactured using PBF-LB technology. It ensures levels of ductility adequate to mitigate the risk of brittle fracture during service-life, while promoting a stable microstructural state free of residual stress, which is essential for long-term reliability.

## Figures and Tables

**Table 2:** Surface roughness parameters (Ra and Rz) and defect percentage measured in the two sections examined.

Plane	Ra [μm]	Rz [μm]	Defect Percentage [%]
*xz*	9.3 ± 1.1	49.9 ± 3.7	0.15%
*xy*	4.4 ± 0.7	24.6 ± 3	0.07%

**Table 3:** Electrochemical corrosion parameters (corrosion potential, E_corr_; corrosion current density, i_corr_; and corrosion rate, CR) for samples in the three investigated conditions.

	E_corr_ [V vs. Ag/AgCl]	i_corr_ [A/cm^2^]	Corrosion Rate [mm/Year]
As-Built	−0.522 ± 0.15	4.46 × 10^−6^ ± 1.12 × 10^−7^	0.038
HT1_500 °C	−0.553 ± 0.18	8.08 × 10^−7^ ± 3.25 × 10^−8^	0.007
HT2_800 °C	−0.538 ± 0.13	1.21× 10^−5^ ± 1.46 × 10^−6^	0.104

## Data Availability

The original contributions presented in this study are included in the article. Further inquiries can be directed to the corresponding author.
